# Discovery of the natural product 3',4',7,8-tetrahydroxyflavone as a novel and potent selective BRD4 bromodomain 2 inhibitor

**DOI:** 10.1080/14756366.2021.1906663

**Published:** 2021-04-06

**Authors:** Jiao Li, Wei Zou, Koukou Yu, Bing Liu, Weifeng Liang, Lisha Wang, Yin Lu, Zequn Jiang, Aiyun Wang, Jiapeng Zhu

**Affiliations:** a School of Medicine & Holistic Integrative Medicine, Nanjing University of Chinese Medicine, Nanjing, Jiangsu, China; b Jiangsu Key Laboratory for Pharmacology and Safety Evaluation of Chinese Materia Medica, School of Pharmacy, Nanjing University of Chinese Medicine, Nanjing, Jiangsu, China; c Department of Medicinal Chemistry, PharmaBlock Sciences (Nanjing), Inc., Nanjing, China

**Keywords:** Bromodomain-containing protein 4, natural product, 3',4',7,8-tetrahydroxyflavone, inhibitor, co-crystal structure

## Abstract

Bromodomain-containing protein 4 (BRD4) binds acetylated lysine residues on the N-terminal tails of histones through two bromodomains (BD1 and BD2) to regulate gene transcription. Inhibiting one or both of bromodomains resulted in different phenotypes, suggesting BD1 and BD2 may have different functions. Here we report the characterisation of a natural product 3',4',7,8-tetrahydroxyflavone as a novel and potent selective BRD4 inhibitor. The compound is 100-fold more selective for BRD4-BD2 (IC50 = 204 nM) than BRD4-BD1 (IC_50_=17.9 µM). Co-crystal structures show 3',4',7,8-tetrahydroxyflavone binds to the acetylated lysine binding pocket of BRD4-BD1 or BRD4-BD2, but establishes more interactions with BRD4-BD2 than BRD4-BD1. Our data suggest 3',4',7,8-tetrahydroxyflavone as a potent selective inhibitor of BRD4-BD2 with a novel chemical scaffold. Given its distinct chemical structure from current BRD4 inhibitors, this compound may open the door for a novel class of anti-BRD4 inhibitors by serving as a lead compound.

## Graphical Abstract

**Figure:**
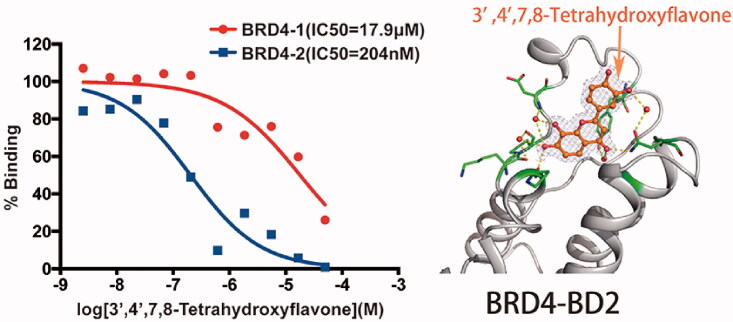

## Introduction

Bromodomain-containing proteins (BRDs) recognise acetylated lysine (KAc) residues on the N-terminal tails of histones to restructure chromatin and regulate transcription^1^^,^^2^. In the human proteome, there are 61 bromodomains located in 46 proteins that can be classified into eight families, one of which is the Bromodomain and Extra C-terminal domain (BET) family^3^. Bromodomain-containing protein 4 (BRD4), a member of the BET family proteins, recruits transcriptional elongation factor complexes (P-TEFb) and facilitates RNA polymerase II-mediated transcription in eukaryotes^1^^,^^4^.

BET proteins play an important role in pathogenesis including cancer and immune diseases due to their ability in modulating gene expression. To date, BRD4 is the most extensively studied BET family protein and has attracted great interest as a drug target. As other members of the BET family (BRD2, BRD3, and the testis-ovary specific BRDT), BRD4 has two tandem bromodomains (BD1 and BD2) and an extra C-terminal domain (ET) with a high sequence conservation through evolution^5^. Both BRD4-BD1 and BD2 interact with acetylated lysine residues in histone and non-histone proteins to regulate a variety of cellular process including transcription, DNA replication, cell cycle progression and others. BD1 and BD2 have been reported to recognise distinct sets of acetylated lysines and may exert different function through transcriptional regulation^6^. BD1 preferentially binds acetylated lysines 5 and 8 of histone H4 tail peptides, while BD2 has more affinity for acetylated lysines 18 and 23 of histone H3 tail peptides^7^. The same bromodomains, BD1 or BD2, are more conserved across the BET family than different bromodomains in the same protein^8^. For example, the sequence of BRD4-BD1 is more similar with BRD2-BD1 than BRD4-BD2. Thus, it is easier to design bromodomain-selective inhibitors than protein-selective inhibitors. Inhibitors that bind BD1 and BD2 with equal affinities (pan-BET inhibitors) are dose-limiting toxic, which cause a reduced number of thrombocytes in the blood (thrombocytopenia) and symptoms of gastrointestinal toxicity^9^^,^^10^. To date, there are a few bromodomain-selective inhibitors reported. RVX-208, a derivative of the plant polyphenol resveratrol, prefers binding to second bromodomains of BET family proteins and increases plasma levels of the high-density lipid protein ApoA1^11^. ABBV-744, a potent and selective inhibitor of the BD2 of BET family proteins showed anti-proliferative activity in cell lines of acute myeloid leukaemia and prostate cancer with fewer platelet and gastrointestinal toxicities^12^. A recent study suggested that BD1 inhibitors behaved the same as pan-BET inhibitors in cancer models, whereas BD2 inhibitors were predominantly effective in models of inflammatory and autoimmune diseases^13^.

Most of the reported BRD4 inhibitors are derived from several core chemical scaffolds including azepines, 3,5-dimethylisoxazoles, pyridones, triazolopyrazines, tetrahydroquinolines, 4-acyl pyrroles and 2-thiazolidinones^14^. Here, we report 3′,4′,7,8-tetrahydroxyflavone (henceforth referred to as **3478**), a natural product that exists in the heartwood of *Acacia burkittii* and *Acacia acuminata*^15^, as a new inhibitor of BRD4 with a novel chemical scaffold. Natural products play an important role in drug discovery, especially in cancer treatments. They exhibit vast chemical diversity, distinct chemical scaffolds and high degrees of stereochemistry that enable them to provide hits even against difficult screening targets, such as protein–protein interactions^16^. The additional advantage of natural products is their “metabolite-likeness” property that enables cell permissibility^17^. In this study, we provide inhibitory activity assays and structural and functional studies to demonstrate that **3478** is a potent selective inhibitor of BRD4–BD2 with a novel chemical scaffold and new binding mode to BRD4 bromodomains, and by suppressing c-Myc expression, **3478** reduced the human acute myeloid leukaemia (AML) MV4‑11 cell growth *in vitro* and xenografted tumour growth in mice without affecting body weight. These data suggest that **3478** can be further explored as a novel class of anti-BRD4 inhibitors by serving as a lead compound.

## Materials and methods

### Chemistry

**3478** (purity (HPLC): 99%) was purchased from BIOSYNTH® Carbosynth Ltd (Compton, UK). The pan-BET inhibitor JQ1 (purity (HPLC) > 97%) was purchased from Changchun Sanbang Pharmaceutical Technology (Changchun, China). The human AML cell line MV4-11 was a kind gift from Professor Tao Lu, China Pharmaceutical University. The A549 and HepG2 cell lines were from American type culture collection (ATCC). The BALB/cJGPT-Foxn 1nu/Gpt mice were purchased from GemPharmatech Co., Ltd. (Nanjing, China). The primary anti-c-Myc antibody, primary anti-BRD4 antibody and secondary goat anti-rabbit IgG H&L antibody were purchased from Abcam (Cambridge, UK).

### AlphaScreen compound–protein interaction assay

The AlphaScreen assays were performed by Reaction Biology Corp. (Malvern, PA). Recombinant Human N-terminal Histagged BRD4 BD1 and BD2 were expressed in *Escherichia coli*. The C-terminal-biotinylated histone H4 peptide (residues 1–21) was used as ligand in the assay. In each well of the reaction plate, a mixture of BRD4 BD1 or BD2 and one individual compound in a reaction buffer containing 50 mM HEPES, 100 mM NaCl, 0.05% CHAPS, 0.1% BSA and 1.0% DMSO (pH7.5) was added and pre-incubated for 30 min. C-Terminal-biotinylated histone H4 peptide was added to each well and incubated for 30 min at room temperature with gentle shaking. Then streptavidin-coated donor beads and AlphaScreen Ni acceptor beads (PerkinElmer, Waltham, MA, USA) were added and the plate was sealed and incubated in the dark for 60 min. When excited by 680 nm laser, donor beads generated singlet oxygens, which diffused to acceptor beads and was converted to light signal between 520 and 620 nm that was recorded with an AlphaQuest®-HTS Microplate reader (PerkinElmer, Waltham, MA, USA). IC_50_ values and curve fits were obtained using GraphPad Prism 7.0 (San Diego, CA, USA).

### AlphaScreen counter assay

A mixture of 50 nM of His-tagged biotin (Met-His-His-His-His-His-His-Ser-Ser-Gly-Val-Asp-Leu-Gly-Thr-Lys-(Biotin)), streptavidin-coated donor beads and Ni-chelate acceptor beads were distributed into 96-well plate. Both of beads bound to the His-tagged biotin. Then **3478** was added in a series of dilution to test the compound’s interference of binding between the streptavidin-coated donor beads and the AlphaScreen Ni-chelate acceptor beads with the His-tagged biotin, by monitoring light signal between 520 and 620 nm.

### Cell proliferation assays

The human acute myelomonocytic leukaemia cell line (MV4-11) was grown in Iscove's Modified Dulbecco's Medium (IMDM) (Gibco, Grand Island, CA). Human lung adenocarcinoma cell line (A549) and human liver carcinoma cell line (HepG2) were grown in Dulbecco's Modified Eagle Medium (DMEM) (Gibco, Grand Island, CA, USA). Human gastric cancer cell line (MKN45) was grown in RPMI-1640 (Gibco, Grand Island, CA, USA). All mediums were supplemented with 10% foetal bovine serum (FBS) (Gibco, Grand Island, CA, USA) and 1% penicillin/streptomycin (Hyclone, Lohan, UT). All cell lines were cultured and maintained in an atmosphere consisting of CO_2_ (5%) and room air (95%) at 37 °C. To determine MV4-11 cell proliferation, approximately 1 × 104 MV4-11 cells were seeded into each well of a 96-well plate and cultivated in 100 µL of culture media supplemented with 10% FBS. Then in each well different concentration of **3478**, JQ1 (positive control) and DMSO (negative control) were added. After 48 h, 10 µL of the Cell Counting Kit-8 (CCK8) solution (Beyotime, Shanghai, China) was added to each well and incubated for 1 h. Cell densities were measured by a microplate reader (Biotek, Winooski, VT, USA).

### Apoptosis assay

FITC Annexin V Apoptosis Detection Kit I (BD Biosciences, Franklin Lakes, NJ) was used to measure the percentage of apoptotic cells. The exposure of phosphatidylserine on the extracellular side of the cell membrane was quantified by Annexin V-FITC/PI staining. MV4-11 cells were incubated with different concentrations of JQ1 and **3478.** After 24 h and 48 h treatment, the cells were harvested respectively. Then they were washed twice with cold phosphate buffer saline (PBS) and incubated with 5 µL Annexin V-FITC and 5 µL PI at room temperature for 15 min in the dark. Binding buffer (500 µL, 1×) was subsequently added to each tube and the cells were immediately analysed using fluorescence-activated cell sorting (FACS) (BD Accuri C6, Franklin Lakes, NJ, USA).

### Construction of AML xenograft mice model and in vivo tumour suppression experiment

Male BALB/c nude mice aged 6-week old and weighing 18–22 g were purchased from GemPharmatech Co., Ltd. (Nanjing, China). The mice were housed (five per cage) under standard laboratory conditions ((22 ± 2) °C, humidity (50 ± 5)%, light/dark cycle 12/12 h) and maintained in a specific pathogen-free facility. All mice were allowed free access to the normal chow diet and tap water. Ten mice were randomly chosen and reserved as the control group, which were orally administered with 0.1 ml/10g 0.3% Carboxymethylcellulose sodium (CMC-Na) once a day; the rest of mice were each injected with approximately 5 × 10^6^ MV4-11 cells into the right lower extremity and allowed two weeks to establish tumours. The mice were monitored daily for general health, and body weights were measured twice weekly. When the mean tumour volume reached 100 mm^3^, the mice were randomly divided into four experimental groups (*n* = 10): model group (orally administered with 0.1 ml/10g 0.3% CMC-Na), JQ1 group (orally administered with 50 mg/kg JQ1 daily), and **3478** groups (orally administered with 50 and 100 mg/kg **3478** daily). JQ1 and **3478** were dissolved in 0.3% CMC-Na solution. The tumour size was measured with a vernier calliper twice a week and the tumour volume was calculated as 0.5 × *L* × *W*×*H*, where *L*, *W*, and *H* represent the longest dimension, widest dimension, and the highest dimension of the tumour, respectively. Relative tumour volumes were calculated as *V*_t_/*V*_0_ (*V*_t_ and *V*_0_ represent the tumour size of the day of measurement and the day of initial treatment, respectively). Mice were weighed twice a week and considered dead when tumour volume reached 1000 mm^3^ or the mice died during treatment. After 4 weeks of administration, mice were sacrificed by cervical dislocation and xenograft tumours were excised and stripped of non-tumour tissue and weighed. The tumours were bisected: one part was fixed in 10% formalin and paraffin-embedded for immunofluorescence staining; the other part was snap-frozen and stored in liquid nitrogen for Western blotting and quantitative PCR analysis.

All experimental protocols were approved by the Animal Care and Use Committee of Nanjing University of Chinese Medicine (Nanjing, China) and conducted under the Guidelines for the Care and Use of Laboratory Animals (202005A024). Institutional and regional ethic committees approved all procedures above.

### Western blotting

Cell and tumour tissue homogenates were lysed in radioimmunoprecipitation assay buffer (Thermo Fisher Scientific, Waltham, MA, USA) containing protease and phosphatase inhibitors (Roche, Basel, Switzerland, Cat. nos. 04693116001 and 04906837001) on ice for 30 min. After centrifugation at 14,000 rpm for 5 min at 4 °C, the supernatant was collected and the protein concentration was determined using BCA assay. Western blotting was performed as previously described^18^. Briefly, proteins were separated by sodium dodecyl sulphate-polyacrylamide gel electrophoresis and immunoblotted with antibodies against BRD4, c-Myc (Abcam, Cambridge, UK), and GAPDH (Bioworld, Visalia, CA). The protein bands were visualised with enhanced chemiluminescence reagent (Biosharp, Wuhan, China) and the protein levels were quantified by scanning densitometry (Gel Doc‐2000, Bio‐Rad, Hercules, CA, USA).

### Immunofluorescence staining

Immunofluorescence staining was performed as described^19^^,^^20^. Mice were sacrificed and tumour tissues were fixed in 4% PFA, dehydrated, and embedded in paraffin. Embedded samples were sectioned and 5 µm sections were immunostained with the following antibodies. Primary antibodies: Anti-c-Myc antibody (Abcam, Cambridge, UK); secondary antibodies: Goat Anti-Rabbit IgG H&L (Abcam, Cambridge, UK). Nuclei were counterstained by Hoechst (Beyotime, Shanghai, China) at room temperature for 15 min. Pictures were captured with a fluorescence microscope (Zeiss, Jena, Germany).

### Quantitative RT-PCR

SYBR green-based real time quantitative PCR was used to measure the mRNA transcriptional levels. Briefly, total cellular RNA from the lysates of transplanted tumours or cells was extracted with chloroform after addition of Trizol reagent (Thermo Fisher Scientific, Waltham, MA, USA). After precipitated with isopropanol, the RNA fraction was dissolved in DEPC-H_2_O. An aliquot of 5 µg RNA was reverse-transcribed into cDNA with a HiScript II QRT SuperMix for qPCR (+gDNA wiper) kit (Vazyme Biotech Co., Ltd, Nanjing, China). Quantitative RT-PCR was performed using a SYBR Green Master kit (Bio-Rad, Hercules, CA, USA). The primer pairs for RT-PCR are as follows: GAPDH (forward: 5′-GGTTGTCTCCTGCGACTTCA-3′, reverse: 5′-TGGTCCAGGGTTTCTTACTCC-3′), c-Myc (forward: 5′-GGCTCCTGGCAAAAGGTCA-3′, reverse: 5′-CTGCTAGTTGTGCTGATGT-3′). Gene expression levels of the samples were calculated relative to the control using comparative CT method as follows: ΔΔCT = ΔCT_sample_ – ΔCT_control_, fold change = 2^–ΔΔCT^. GAPDH expression was used as the internal control.

### Statistical analysis

Except the data from mice tumour tissue, all *in vitro* data were obtained from at least three repeated experiments and are expressed as the MEAN ± SEM (standard error of the mean). Two-group comparisons were made by the unpaired Student's *t*-test, and multiple comparisons were analysed by one-way analysis of variance (ANOVA)^21^. Differences were considered significant when *p* < 0.05. Quantitative analyses were carried out with GraphPad Prism 7.0 (San Diego, CA, USA).

### Protein expression and purification

The gene encoding BRD4-BD1 with an N-terminal His-tag and a thrombin cleavage site was codon optimised, synthesised and subcloned into the pET-28a expression plasmid. The plasmids were transformed into BL-21 cells and the cells were grown in LB media at 37 °C until OD_600_ reached 0.6. Then the cells were induced with 0.2 mM IPTG overnight at 22 °C. The cells were collected and resuspended in Buffer A containing 500 mM NaCl, 5 mM imidazole, 5% *w/v* glycerol and 50 mM HEPES (pH 7.5), and disrupted using a high pressure homogeniser (ATS Engineering), followed by 10 min of centrifugation at 20,000 *g* to remove cell debris. The supernatant was loaded onto a Ni-NTA (Qiagen, Valencia, CA, USA) column pre-equilibrated with buffer A. The column was washed with 25 ml of the same buffer containing 50 mM imidazole in order to remove non-specific bound protein. His-tagged BRD4 BD1 was eluted with buffer B (500 mM NaCl, 250 mM imidazole, 5% *w/v* Glycerol, 50 mM HEPES, pH 7.5). The peak fractions from the Ni-NTA column were pooled and cleaved with thrombin overnight at 4 °C. The cleaved BRD4 BD1 was further purified with a Superose 12 gel filtration column (GE Healthcare, Chicago, IL, USA) and concentrated to 7 mg/mL. The expression and purification of BRD4 BD2 was essentially the same as BD1, except a TEV cleavage site was inserted between the His-tag and the target gene, and the Ni-NTA column purified BD2 protein was cleaved with TEV protease.

### Crystallisation, data collection and processing

Crystallisation was performed by using the sitting drop vapour diffusion method. For BD1, 1 µL solution containing 7 mg/mL of protein in 20 mM Tris and 50 mM NaCl (pH 8.0), was mixed with 1 µL of well solution containing 4.0 M sodium formate and 200 mM NDSB-201, with microseeding of native BRD4 BD1 crystal seeds. The crystallisation drop was incubated against 50 µL of well solution at 295 K. Crystals appeared in 12 h and reached the maximum size in 2 days. For BD2, 1 µL protein solution containing 10 mg/mL of protein in 20 mM Tris and 50 mM NaCl (pH 8.0), was mixed with 1 µL of well solution containing 20% *v/v* polyethylene glycol monomethyl ether 2000 and 100 mM Tris (pH 8.5) and incubated against 50 µL of well solution at 295 K. BD2 crystals appeared in 12 h and reached the maximum size in 24 h. Protein-compound complex crystals were obtained by soaking the native crystals in a solution containing the same well solution supplemented with some granules of the compound.

BD1-**3478** crystals were transferred to a cryoprotectant solution comprising 4.0 M sodium formate and 20% glycerol, and BD2-**3478** crystals were transferred to a cryoprotectant solution comprising 27% *v/v* polyethylene glycol monomethyl ether 2000 and 100 mM Tris (pH 8.5), then the cryo protected crystals were flash-frozen in liquid nitrogen. X-ray data of each protein − compound complex were collected from a single crystal at 100 K at BL17U1 of Shanghai Synchrotron Radiation Facility. Images were processed with Mosflm^22^. BD1 crystals belong to space group P2_1_2_1_2_1_ and BD2 crystals belong to the space group P22_1_2_1_ (Table 1). The structures of protein − compound complexes were solved by molecular replacement implemented in Phaser^23^. Coordinates of BD1 (PDB ID code 4PCE) and BD2 (PDB ID code 6C7Q) were used as the original search models. The structure models of the compound were manually fit in the electron density using COOT^24^. The built models were refined by the program PHENIX. Figures of protein structures were created with Pymol (http://www.pymol.org). The coordinates for the models of BRD4-BD1 and BRD4-BD2 have been deposited in the Protein Data Bank, https://www.rcsb.org (PDB ID codes 7C2Z and 7C6P). Authors will release the atomic coordinates and experimental data upon article publication.

**Table 1.** Data collection statistics for crystals of BD1-**3478** and BD2-**3478** complexes.

Property	Value	
	BD1**–3478** complex	BD2**–3478** complex
Space group	P 2_1_2_1_2_1_	P 2 2_1_ 2_1_
Cell constants a, b, c	32.23 Å 47.22 Å 79.56 Å	33.59 Å 63.01 Å 70.50 Å
Resolution (Å)	40.61–1.30 (1.32–1.30)	35.25–1.73 (1.76–1.73)
% Data completeness	86.0 (87.1)	99.9 (100)
*R*_merge_	0.089 (0.120)	0.079 (0.138)
<I/σ(I)>^a^	4.4 (1.0)	6.3 (1.0)
CC_1/2_	0.991 (0.533)	0.985 (0.415)
Redundancy	3.0 (3.5)	3.9 (5.4)
*R*_work_/*R*_free_	0.1883/0.1992	0.1893/0.2216

Values shown in parentheses are for the highest resolution shell and data are from a single crystal only.

^a^Intensities estimated from amplitudes.

## Results

### AlphaScreen identified 3478 as a potent selective inhibitor of BRD4 BD2

**Figure 1.**
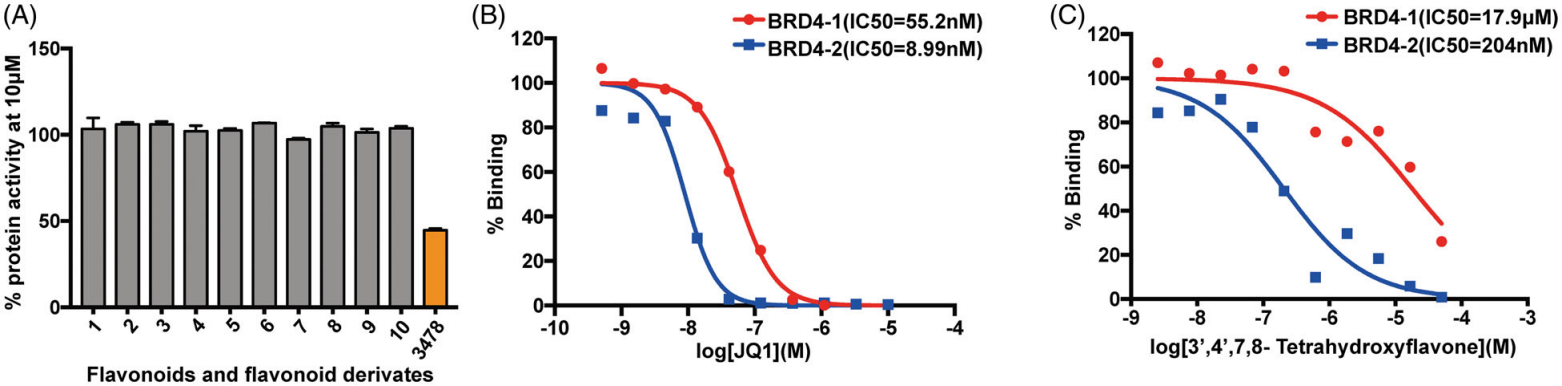
AlphaScreen binding assays. (A) Inhibitory activities of 10 μM different compounds against BRD4-BD1. (B) 10 dose AlphaScreen assays of JQ1 against BRD4-BD1 and BD2. (C) 10 dose AlphaScreen assays of **3478** against BRD4-BD1 and BD2.

Of the 11 compounds (flavonoids and derivatives) tested with AlphaScreen assay at 10 µM, only **3478** showed apparent inhibition against BRD4-BD1 (Table 2 and Figure 1(A)). Then **3478** was further tested with 10 dose IC_50_ AlphaScreen assay against BRD4-BD1 and BD2 to determine the accurate inhibitory activities. Simultaneously, AlphaScreen counter assay was also performed to eliminate the false positive. Remarkably, **3478** was ∼100-fold selective for BRD4-BD2 (IC_50_=204 nM) than BRD4-BD1 (IC_50_=17.9 µM) (Figure 1(C)). The positive control JQ1 showed no significant selectivity (IC_50_=55.2 nM for BRD4-BD1 and IC_50_=8.99 nM for BRD4-BD2) (Figure 1(B)). Meanwhile, the AlphaScreen counter assay showed that **3478** did not produce notable false positive results (Table 3).

**Table 2.** Inhibitory activities of flavonoids and derivatives.

No.	Compound	Chemical structure	(% protein activity in the presence of 10 μM compound)
1	Naringenin		103.50
2	Taxifolin		106.08
3	Hesperetin		106.01
4	Genistein		102.01
5	Flavopiridol		102.56
6	6-Phenyl-3,4-dihydro-2(1h)-one		106.80
7	1N-Methyl-6-phenyl-3,4-dihydro-2(1h)-one		97.35
8	1N-Methyl-7-phenyl-3,4-dihydro-2(1h)-one		104.97
9	7-Phenyl-3,4-dihydro-2(1h)-one		101.44
10	3′,4′,7,8-Tetrahydroxyflavanone		103.78
**3478**	3′,4′,7,8-Tetrahydroxyflavone		44.68

**Table 3.** AlphaScreen counter assay.

Compound	Concentration (M)	% Binding
3478	5.00 × 10^-5^	98.39
	1.67 × 10^-5^	96.40
	5.56 × 10^-6^	97.72
	1.85 × 10^-6^	97.84
	6.17 × 10^-7^	103.76
	2.06 × 10^-7^	95.81
	6.86 × 10^-8^	102.51
	2.29 × 10^-8^	101.85
	7.62 × 10^-9^	102.56
	2.54 × 10^-9^	101.61
DMSO^a^	–	98.67

^a^The concentration of DMSO control was 1% *v/v*.

### 3478 Binds to the ligand binding pocket of BRD4-BD1 or BRD4-BD2 and His433 of BRD4-BD2 is responsible for the selectivity

**Figure 2.**
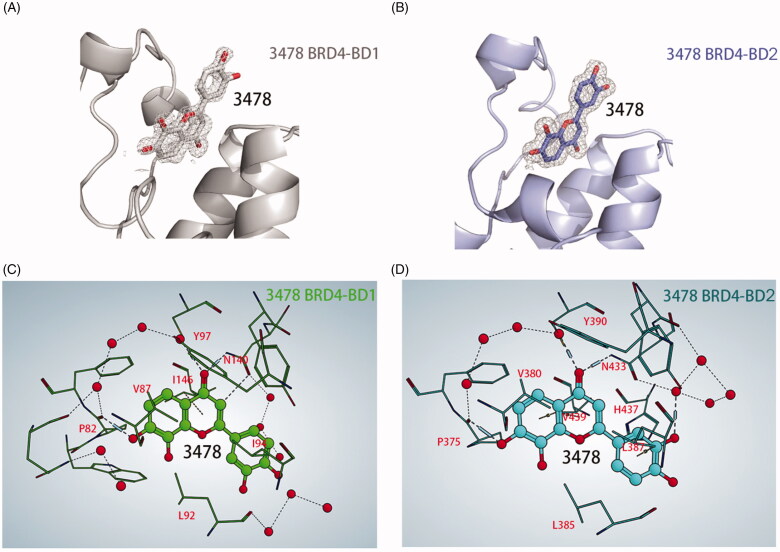
Cartoon representations of the co-crystal structures of BRD4-BD1 (A) and BRD4-BD2 (B) in complex with **3478 (**stick representation**)**. 2Fo-Fc maps are contoured at 2.0 σ. BRD4-BD1 is coloured in grey and BRD4-BD2 in purple. Interactions of **3478** with BRD4-BD1 (C) and BRD4-BD2 (D). **3478** is shown in ball and stick with the oxygen atoms in red, and waters are shown as red spheres. The blue cylinder lines represent the H-bonds of the ligand with the protein and the structured waters, and the blue thin lines represent the H-bonds of water with water or water with protein. MOE 2019.01 (https://www.chemcomp.com/) analysed the H-bond network and generated the figures (C, D).

To better understand the mechanism of different affinity activities of **3478** to BRD4-BD1 and BD2, we performed X-ray structures of **3478** in complex with BRD4-BD1 and BRD4-BD2 which were determined at resolution of 1.30 Å and 1.73 Å (Table 1), respectively. **3478** binds very similarly to BRD4-BD1 and BRD4-BD2 in the binding pocket (Figure 2). In both structures, the carbonyl group of **3478** forms strong H-bonds with an asparagine residue (BRD4-BD1: N140, BRD4-BD2: N433), and a water molecule near the tyrosine residue (BRD4-BD1: Y97, BRD4-BD2: Y390). 7-hydroxy of **3478** forms a third H-bond with the main chain carbonyl group of a proline residue (BRD4-BD1: P82, BRD4-BD2: P375). These three H-bonds fix the 2,3‐dihydro‐1‐benzopyran‐4‐one ring tightly in the binding pocket. In addition, hydrophobic residues V87, I146 and I94 (BRD4-BD1) or V380, V439, and L387 (BRD4-BD2) formed strong hydrophobic interaction with **3478 (**Figure 2(C,D)).

In the crystal of BD1-**3478** complex, the weak electron density of the phenyl group of **3478** suggests a weak interaction of this phenyl group with BRD4-BD1 (Figure 2(A)). In contrast, its density is well defined in BD2-**3478** structure (Figure 2(B)), suggesting a fixed conformation and stronger interactions in BRD4-BD2. **3478** forms one additional H-bond with the water molecule near N433, and likely establishes hydrophobic interaction with His437, as NH of His437 points to two carbon atoms in its phenyl ring. The corresponding position of His433 in BRD4-BD1 is an aspartic acid (D144) whose side chain is too distant from **3478** to establish effective interaction. In a recently published paper, His437 of BD2 is exploited to design inhibitors with selectivity for BD2^12^.

### BRD4 is highly overexpressed in AML cancer patients and its level is an indicator of prognosis

**Figure 3.**
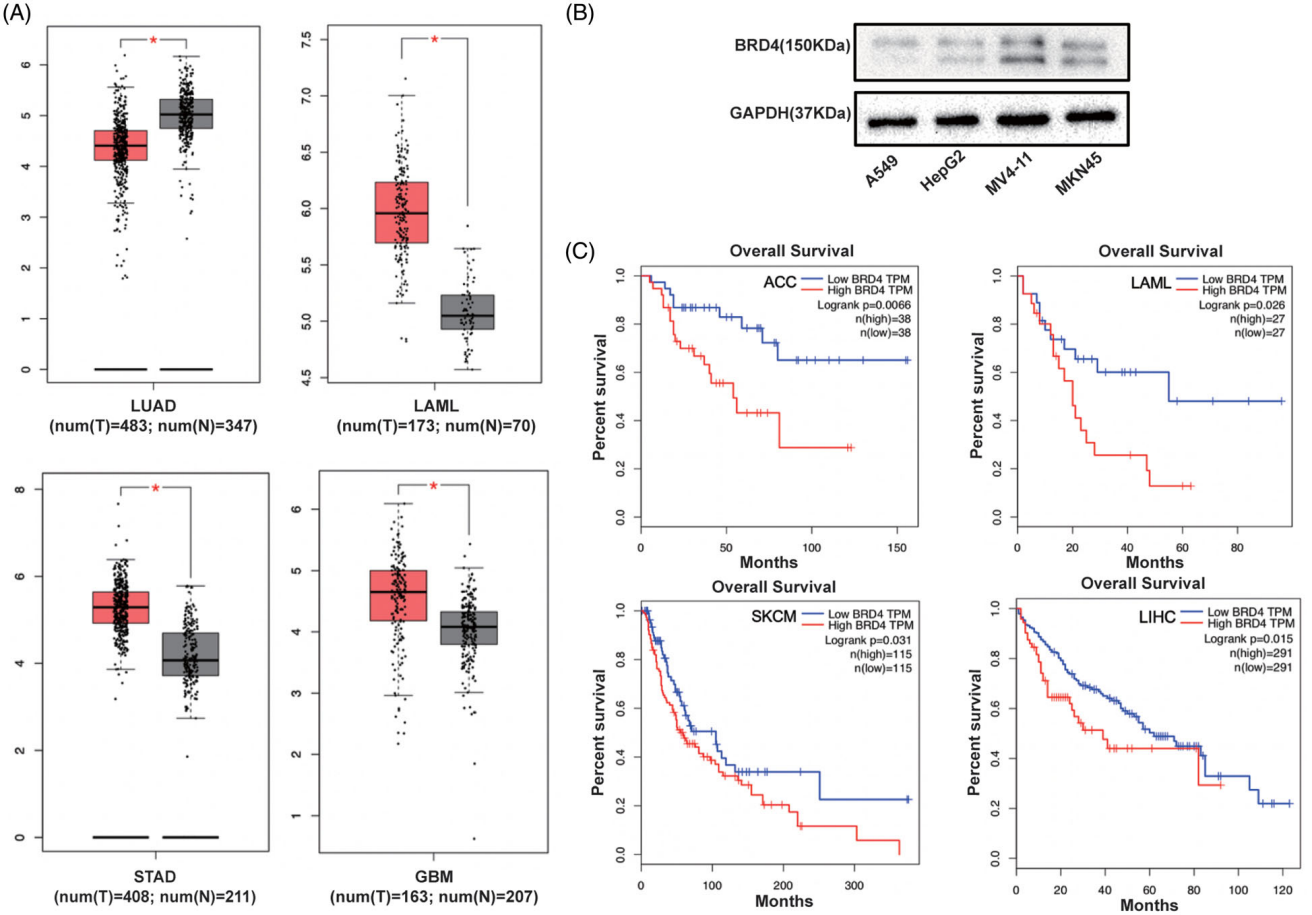
(A) The BRD4 expression levels in four cancers, detected by TIMER (*p < 0.05). LUAD, LAML, STAD and GBM stand for lung adenocarcinoma, acute myeloid leukaemia, stomach adenocarcinoma and glioblastoma multiforme, respectively. (B) Western blotting analysis of BRD4 expression in A549, HepG2, MV4-11, and MKN45 cell lines. (C) Comparison of Kaplan–Meier survival estimates with respect to BRD4 expression levels in different cancers. Low BRD4 expression levels show favourable overall survival (OS) in adrenal cortical carcinoma (ACC), LAML, skin cutaneous melanoma (SKCM) and liver hepatocellular carcinoma (LIHC), according to the Kaplan–Meier plotter database.

By querying Cancer Genome Atlas (TCGA) database (http://gepia.cancer-pku.cn), we found that in acute myeloid leukaemia (LAML) patients, the mRNA expression level of BRD4 in tumours was significantly higher than in normal controls (Figure 3(A)). Consistently, we observed higher levels of BRD4 protein abundance in MV4-11 (AML) and MKN45 (gastric cancer) compared with A549 (lung cancer) and HepG2 (liver cancer) cells (Figure 3(B)). In addition, an inverse correlation between BRD4 expression and overall survival rate in LAML and ACC cohorts were observed, suggesting that lower BRD4 expression may be associated with a favourable prognosis (Figure 3(C)).

### 3478 Inhibited MV4-11 cell proliferation and induce apoptosis

**Figure 4.**
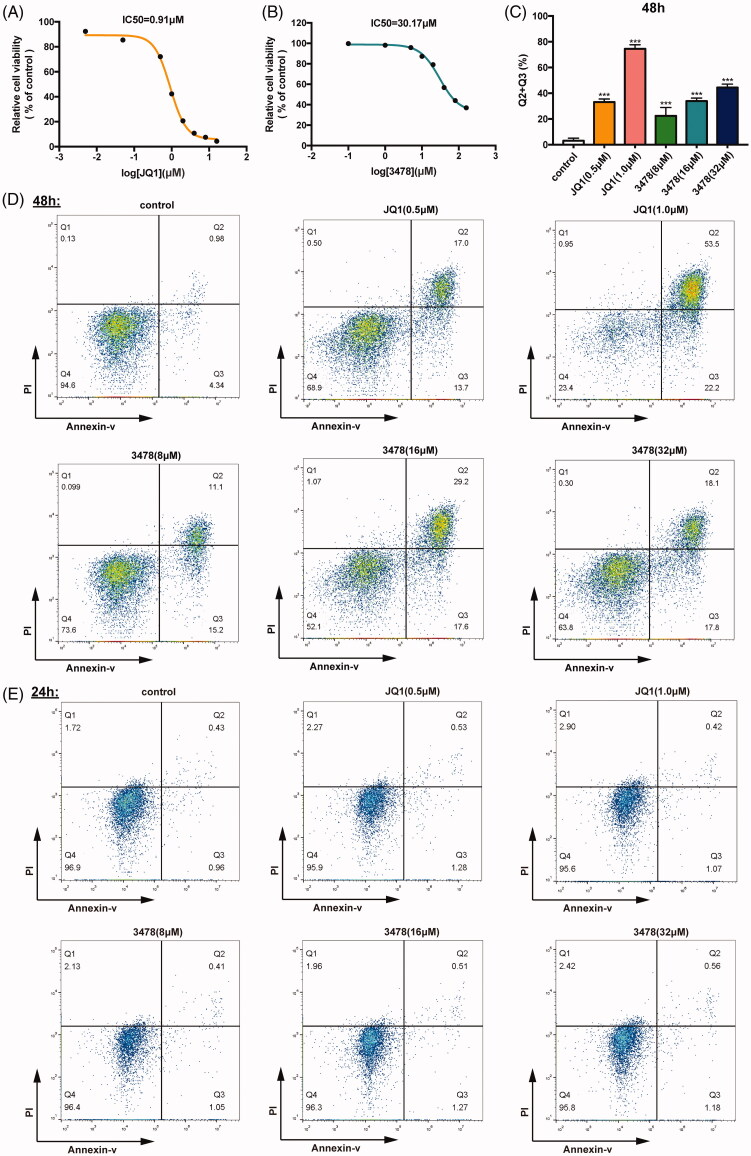
**3478** inhibited MV4-11 cells growth. MV4-11 cells were treated with JQ1 (A) or **3478** (B) for 48 h and analysed by CCK8 kits. The IC_50_ values were calculated using GraphPad Prism 7.0. (C) The percentages of apoptotic cells after 48 h treatment (the lower-right quadrant of the FACS histograms (percentage of early apoptotic cells) and the upper-right quadrant (percentage of late apoptotic cells)) are shown. All results were representative images from three experiments. The MV4-11 cells were exposed to different concentrations of JQ1, **3478** or culture medium for 48 h (D) and 24 h (E), and stained with Annexin V-FITC and PI for apoptosis measurement by flow cytometry.

JQ1 was used as the positive control as it was one of the most studied BRD4 inhibitors that shown anti-tumour potential for both blood cancers and solid tumours^25^^,^^26^. CCK8 assays showed that JQ1 and **3478** inhibited MV4-11 cell growth in a dose-and time dependent manner. IC_50_ of JQ1 and **3478** were 0.91 µM (Figure 4(A)) and 30.17 µM (Figure 4(B)) after 48 h, respectively. FACS analysis of double-stained MV4-11 cells with Annexin V-FITC/PI showed that JQ1 or **3478** treatment for 48 h caused significantly higher apoptosis rates compared with untreated control cells (Figure 4(C,D)), but for 24 h this effect was not significant (Figure 4(E)).

### 3478 Retarded tumour growth in the MV4-11 xenograft mice model

**Figure 5.**
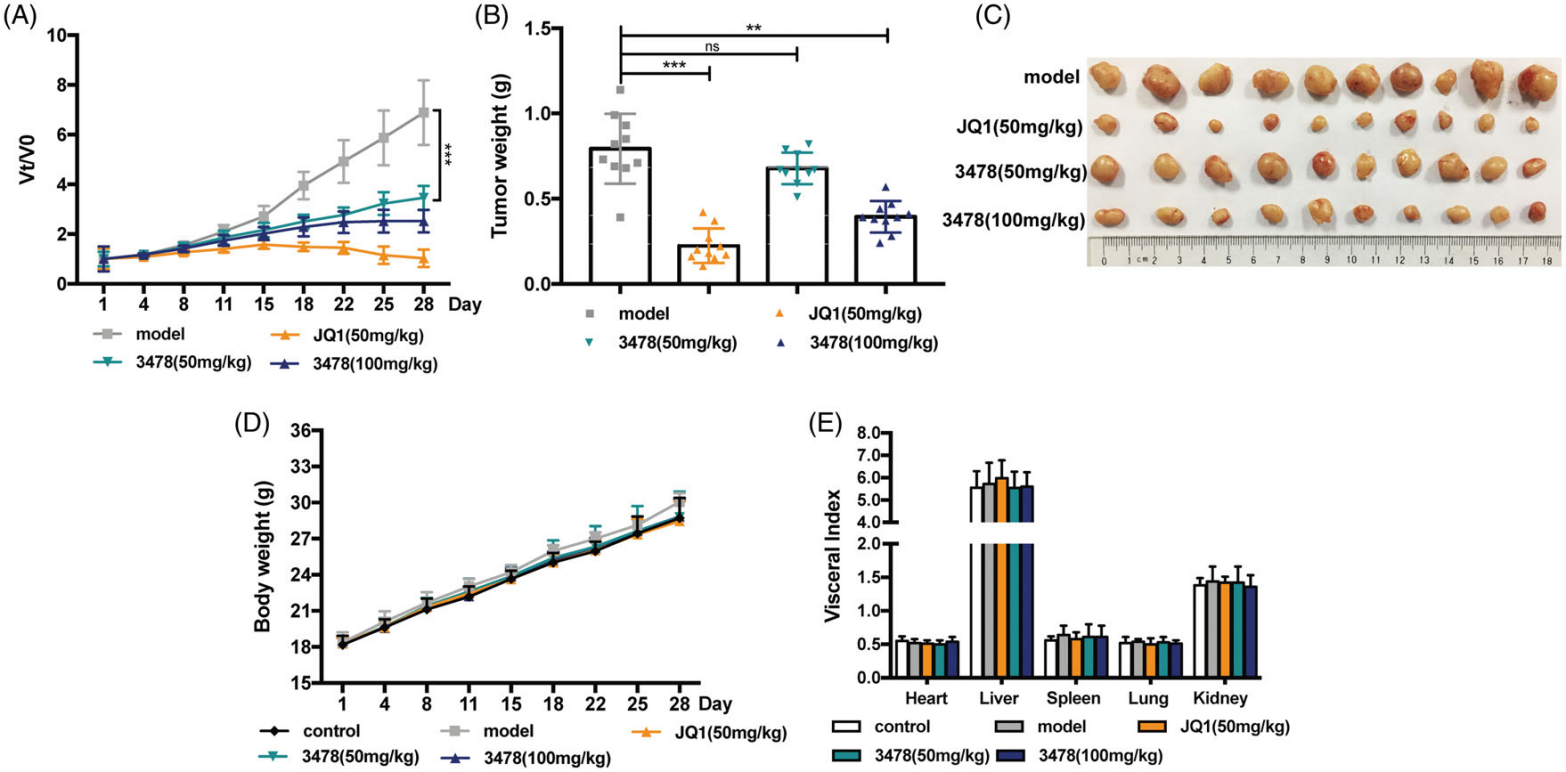
The effect of **3478** on MV4-11-implanted AML tumour *in vivo*. (A) Tumour volumes of mice treated with 0.3% CMC-Na (model), JQ1(50 mg/kg), **3478** (50 mg/kg), and **3478** (100 mg/kg). (B) Weights of tumours from different groups of mice on day 28. (C) Photographs of tumour blocks from different groups on day 28. (D) Body weight changes of mice in different groups at 1, 4, 8, 11, 15, 18, 22, 25, 28 day time points. (E) The visceral index of mice from different groups on day 28 compared with the model group: *p < 0.05, **p < 0.01, ***p < 0.001.

To evaluate the anti-AML efficacy of **3478** *in vivo*, nude mice bearing subcutaneous MV4-11 xenografts were treated with 50 mg/kg or 100 mg/kg **3478** once a day for 4 weeks and compared with negative (0.3% CMC-Na treatment daily) and positive (50 mg/kg JQ1 treatment daily) controls. The results showed that **3478** effectively inhibited tumour progression (Figure 5(A–C)). On day 28, the relative tumour volume was 6.89, 1.03, 3.46 and 2.52 for mice treated with 0.3% CMC-Na, 50 mg/kg JQ1, 50 mg/kg **3478** and 100 mg/kg **3478**, respectively (Figure 5(A)), as shown in Figure 5(C). The average tumour weight on day 28 of 50 mg/kg and 100 mg/kg **3478** dose groups were 0.68 g and 0.39 g, while it was 0.79 g for model group and 0.22 g for JQ1 group (Figure 5(B)). In addition, **3478** caused no notable side effects indicated by body weight and visceral index (Figure 5(D,E)).

### 3478 Suppressed AML progression maybe through regulating BRD4/c-Myc signalling pathway

**Figure 6.**
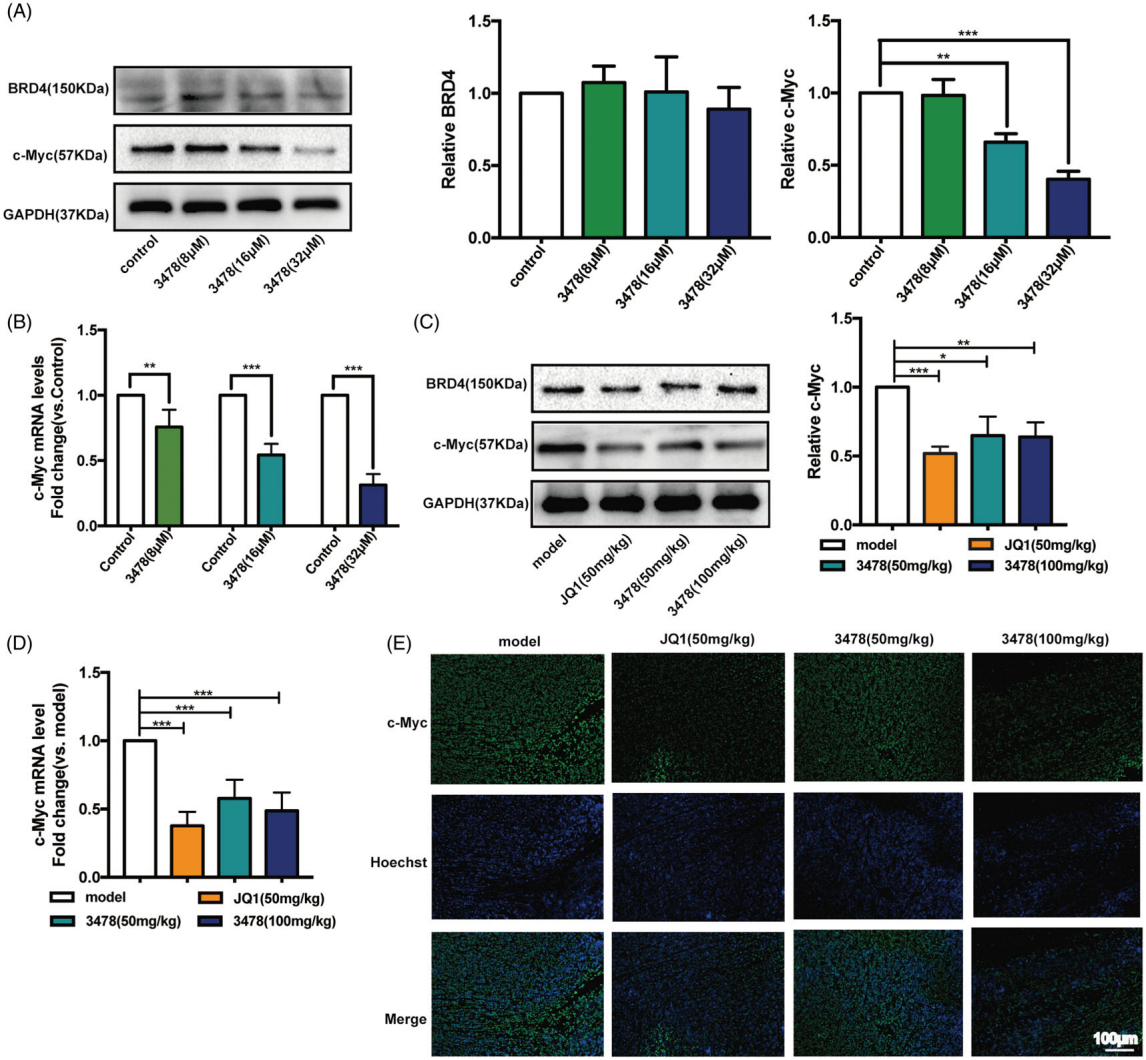
Compound **3478** downregulated the c-Myc both in MV4-11 cells and MV4-11 xenograft tumours through BRD4/c-Myc signalling pathway. (A) The protein expression of BRD4 and c-Myc after **3478** treatment for 24 h were measured by Western blotting. Quantification of protein level was normalised to GAPDH using densitometry. (B) The mRNA level of c-Myc in MV4-11 cells after **3478** intervention for 24 h was determined by real-time qPCR. GAPDH was used as the loading control. (C) The protein expression of BRD4 and c-Myc in MV4-11 xenograft tumours in the different groups were measured by Western blotting. Quantification of protein level was normalised to GAPDH using densitometry. (D) The mRNA level of c-Myc in MV4-11 xenograft tumours in the different groups were determined by RT-qPCR. GAPDH was used as the loading control. (E) Immunofluorescence analysis of c-Myc localisation and expression in MV4-11 transplanted tumour tissues in different groups (100×, scale bar represented 100 μm). Compared with control or model group: *p < 0.05, **p < 0.01, ***p < 0.001. Data in panels represented means ± SD from 10 animals. Independent experiments were performed in triplicate.

C-Myc is a downstream target activated by BRD4, which affects cell proliferation. To identify the signalling pathway responsible for **3478**-induced inhibition of cell growth, we performed Western blotting and RT-qPCR assays after 24 h treatment to examine the mRNA and protein expression levels of related proteins *in vitro*. Our results showed that the level of BRD4 protein was not influenced by **3478** treatment (Figure 6(A)). As expected, **3478** down-regulated c-Myc expression at both mRNA and protein levels as JQ1 did^27^ (Figure 6(A,B)). Consistent with the results *in vitro*, **3478** treatment reduced c-Myc expression at both mRNA and protein levels in MV4-11 xenograft mice (Figure 6(C,D)). Immunofluorescence assay of tumour samples also showed that **3478** treatment inhibited c-Myc expression in nucleus and cytoplasm (Figure 6(E)). Collectively, these results indicate that **3478** targets BRD4-driven oncogenic pathways with increased c-Myc expression.

## Discussion

BRD4 is the most extensively studied BET family member, which is the epigenetic reader of histone code. It recruits transcriptional regulator complexes to chromatin and binds to acetylated lysine (KAc) residues on the N-terminal tails of histones to activate RNA polymerase II. Due to its central role in cell proliferation, apoptosis and transcription, BRD4 is considered a promising drug target for a number of human diseases including cancer, inflammation and cardiovascular diseases. Inhibiting BRD4 displays efficacy against diseases especially cancer and inflammation. Therefore, discovery of high potency and low toxic BRD4 inhibitors attracts huge interest from medical chemists.

Flavonoids are a kind of ubiquitous natural products in plants and essential active ingredients of many medicinal plants. They have the characteristics of broad biological activity, high efficiency and are generally considered safe, with good prevention and cure effects on various types of tumours^28^. Previous studies show flavonoids can inhibit occurrence and development of cancer in various aspects, including the inhibition of aerobic glycolysis, the promotion of apoptosis, the retardation of cell cycle, the suppression of invasion and migration, the induction of DNA damage, and the inhibition of aromatase and microtubule production^29^.

**Figure 7.**
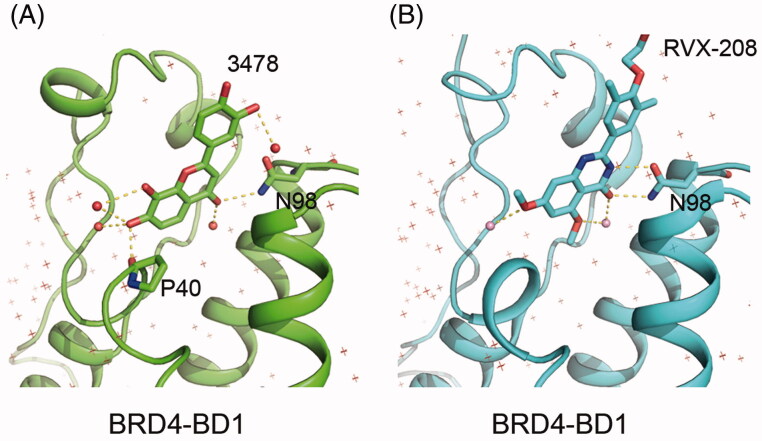
Comparison between BRD4-BD1 in complex with **3478** (A) and RVX-208 (B). Polar contacts were found with Pymol. **3478** is hydrogen-bonded with P40–N98 and other 5 water molecules (total 7 hydrogen bonds) while RVX-208 is hydrogen-bonded with N98 and other two water molecules (total 5 hydrogen bonds).

In this study, we discovered 3′,4′,7,8-tetrahydroxyflavone, a natural flavonoid found in *Acacia burkittii* and *Acacia acuminata* heartwoods^15^, can potently inhibit BRD4-BD2 with ∼100 folds selectivity of BRD4-BD1. The inhibitory activity to BRD4 is not a common feature of flavonoid, as the other 10 flavonoids and derivatives showed no apparent inhibition to BRD4-BD1 at the concentration of 10 µM, suggesting the positions of hydroxyl groups are critical to the inhibitory activity. In addition, an aromatic heterocyclic C ring is important for the inhibitory activity, for 3′,4′,7,8-tetrahydroxyflavanone (No. 10 in Table 2), the hydrogenated derivative of **3478**, loses inhibitory activity to BRD4-BD1. The importance of the hydroxyl group positions is confirmed by co-crystal structure of **3478** in complex with BRD4-BD1 and BD2, in which the compound forms hydrogen bonds with the proteins by its hydroxyl group. The crystal structures suggest that the selectivity is mainly due to one extra hydrogen bond and the hydrophobic interaction between Histidine 437 of BD2 and phenyl group of **3478**. Most of the reported BRD4 inhibitors are derived from several core chemical scaffolds and are distinctively different from **3478**. RVX-208 shows some similarities in the scaffold, but crystallographic analysis shows that their binding modes are very different (Figure 7). Thus, the chemical scaffold of **3478** and its binding mode with BRD4 bromodomains are distinctively different from the reported BRD4 inhibitors to date.

To evaluate the efficacy of **3478** *in vivo*, we constructed MV4-11 mouse xenograft model and the results showed that **3478** had a dose-dependent inhibitory activity: at 50 mg/kg dose, the size of tumour was significantly smaller compared with the negative control group, and at 100 mg/kg dose, the size of tumour was comparable to that treated with 50 mg/kg JQ1. No noticeable body weight loss was observed upon the treatment of 100 mg/kg **3478**, suggesting its low toxicity. Western blotting results showed that compound **3478** remarkably reduced the c-Myc expression at mRNA and protein levels, while BRD4 level remained unchanged.

The protooncogene c-Myc is a key regulator of hyperproliferation, cell cycle progression and metastasis, which is essential for lymphoid^30^^,^^31^ and megakaryocytic/erythroid development^32^. The epigenetic regulation at the c-Myc locus plays a critical role in myeloid differentiation and leukaemia. The BRD4 and SWI/SNF complexes regulate c-Myc expression from the distal super-enhancer BDME (BRD4-dependent c-Myc enhancer), 1.7 megabases (Mb) downstream from its transcription start site (TSS^33^^,^^34^). Disrupting the protein–protein interactions between BRD4 and acetyl-lysine can effectively repress the transcription of c-Myc oncogene and c-Myc dependent genes and inhibit the proliferation of cancer cells such as AML, the activated B-cell-like subtype (ABC) of diffuse large B-cell lymphoma (DLBCL)^35–37^, neuro-blastoma^38^ and lung adenocarcinoma^39^. Therefore, BRD4 has become a promising anti-cancer drug target that attracts huge interest in discovering BRD4 inhibitors^40^.

## Conclusion

We describe the discovery of a natural product, 3′,4′,7,8-tetrahydroxyflavone, as a potent inhibitor of BRD4 with a novel chemical scaffold and binding mode to BRD4 with ∼100-fold selectivity for BRD4-BD2 (IC50 = 204 nM). Since the bromodomains of the BET family proteins are highly conserved, presumably it can selectively inhibitor BD2 of other BET proteins. Considering its high potency, selectivity for BD2, its small size (molecular weight = 286.24) and novel chemical scaffold, it is worth to be explored as a lead compound to inspire anti-cancer drug design and to treat other BRD4-related diseases.
